# Multimodality imaging for real-time image-guided left ventricular lead placement during cardiac resynchronization therapy implantations

**DOI:** 10.1007/s10554-019-01574-0

**Published:** 2019-03-07

**Authors:** Odette A. E. Salden, Hans T. van den Broek, Wouter M. van Everdingen, Firdaus A. A. Mohamed Hoesein, Birgitta K. Velthuis, Pieter A. Doevendans, Maarten-Jan Cramer, Anton E. Tuinenburg, Paul Leufkens, Frebus J. van Slochteren, Mathias Meine

**Affiliations:** 1 Department of Cardiology, University Medical Center Utrecht, Heidelberglaan 100, P.O. Box 85500, 3584 CX Utrecht, The Netherlands; 2 Department of Radiology, University Medical Center Utrecht, Utrecht, The Netherlands; 3 Netherlands Hearts Institute, Central Military Hospital Utrecht, Utrecht, The Netherlands; 4 CART-Tech B.V, Utrecht, The Netherlands

**Keywords:** Cardiac resynchronization therapy, Image-guided interventions, Multimodality imaging, Targeted lead placement, Image fusion

## Abstract

This study was performed to evaluate the feasibility of intra-procedural visualization of optimal pacing sites and image-guided left ventricular (LV) lead placement in cardiac resynchronization therapy (CRT). In fifteen patients (10 males, 68 ± 11 years, 7 with ischemic cardiomyopathy and ejection fraction of 26 ± 5%), optimal pacing sites were identified pre-procedurally using cardiac imaging. Cardiac magnetic resonance (CMR) derived scar and dyssynchrony maps were created for all patients. In six patients the anatomy of the left phrenic nerve (LPN) and coronary sinus ostium was assessed via a computed tomography (CT) scan. By overlaying the CMR and CT dataset onto live fluoroscopy, aforementioned structures were visualized during LV lead implantation. In the first nine patients, the platform was tested, yet, no real-time image-guidance was implemented. In the last six patients real-time image-guided LV lead placement was successfully executed. CRT implant and fluoroscopy times were similar to previous procedures and all leads were placed close to the target area but away from scarred myocardium and the LPN. Patients that received real-time image-guided LV lead implantation were paced closer to the target area compared to patients that did not receive real-time image-guidance (8 mm [IQR 0–22] vs 26 mm [IQR 17–46], *p* = 0.04), and displayed marked LV reverse remodeling at 6 months follow up with a mean LVESV change of −30 ± 10% and a mean LVEF improvement of 15 ± 5%. Real-time image-guided LV lead implantation is feasible and may prove useful for achieving the optimal LV lead position.

## Introduction

Cardiac resynchronization therapy (CRT) has had a major beneficial effect on the treatment of patients with symptomatic heart failure, severe left ventricular (LV) dysfunction, and prolonged QRS duration. Nevertheless up to 30–45% of patients do not obtain a clinical or echocardiographic benefit from CRT [1]. Improving CRT response rate has been the main focus of many researchers in the field, whom have demonstrated that improved response can be achieved by targeting optimal pacing sites for LV stimulation [2–4].

LV lead placement in or near an area of myocardial scar worsens outcomes [4–6], while pacing in or near an area of latest mechanical contraction improves both response rate and prognosis after CRT [2, 3]. Still, the fluoroscopic projections used during CRT implantation provide no tissue characteristics, and therefore no information regarding the optimal site for LV pacing. Consequently, LV leads are mostly placed empirically on the posterolateral wall in patients undergoing CRT. However, there is a substantial inter-individual variation regarding the optimal pacing site as a result of myocardial scar regions and diversity in intrinsic electrical activation of the myocardium. Cardiac magnetic resonance (CMR) imaging has been proposed as a promising tool for LV target area identification since it is able to assess myocardial scar tissue with late gadolinium enhancement (LGE) and mechanical dyssynchrony with feature tracking [6]. Yet, LV lead delivery into target areas remains difficult due to left phrenic nerve (LPN) stimulation. And restrictions caused by coronary vein anatomy.

Aforementioned challenges call for additional techniques that offer real-time visualization of optimal pacing sites during CRT device implantation. In the present study, we test the feasibility of a custom-made treatment-guidance platform (CARTBox [7], CART-Tech B.V., Utrecht, The Netherlands) for real-time visualization of scar location, latest contracting area, and LPN position onto live fluoroscopy during CRT implantation procedures.

## Methods

### Study population

Fifteen patients with an indication for CRT according to the current ESC guidelines were prospectively enrolled [8]. Patients with severely impaired renal function (GFR < 30 ml/min/1.73 m^2^), and patients with a contraindication for cardiac magnetic resonance (CMR), as well as patients with persistent atrial fibrillation, were excluded. All subjects gave written informed consent. The study was performed according to the Declaration of Helsinki and was approved by the local institutional review board and ethics committee.

### Study design

In this prospective feasibility study, LV target areas were determined on pre-procedurally acquired CMR and computed tomography (CT) scans using a custom-made platform, CARTBox (CART-Tech B.V., Utrecht, The Netherlands). After LV target area identification, target areas were co-registered with live fluoroscopy during CRT implantations.

**Fig. 1:**
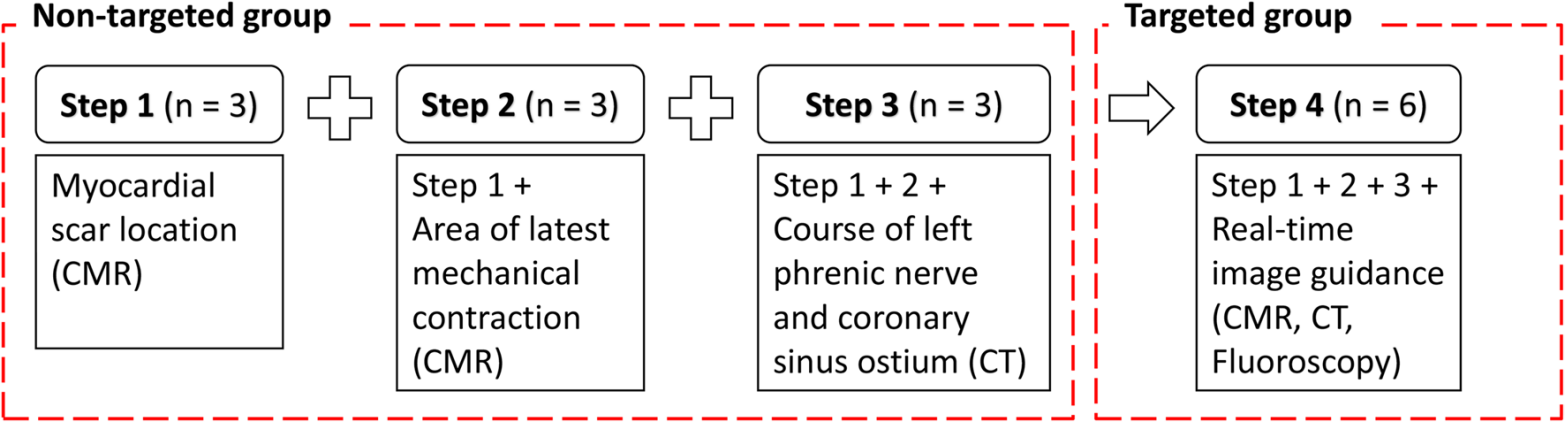
Schematic overview of study. Each step resembles a phase during the study. In step 1 a CMR scan was made to assess the location of myocardial scar tissue (patients 1–3). In step 2 scar identification and contraction timing analysis was performed on CMR images (patients 4–6). In step 3 a CT scan was added to identify the left phrenic nerve and coronary sinus ostium (patients 7–9). In step 1–3 the feasibility of CARTBox was tested for identification and live visualization of the structures. In step 4, steps 1–3 were combined and used for real-time image-guidance of left ventricular lead placement (patients 10–15). *CMR* cardiac magnetic resonance, *CRT* cardiac resynchronization therapy, *CT* computed tomography

The study was conducted in four steps to test the feasibility of the various features of CARTBox in a stepwise approach (Fig. 1). During steps 1–3 (including three patients per step, thus nine patients in total) *specific* tissue characteristics (scar, delayed mechanical activation, the LPN and coronary sinus ostium) (CSO) were identified on a pre-procedural CMR or CT scan. Based on the location of scar, delayed activation, and the LPN, a target area for LV lead delivery was chosen. Importantly, LV lead target area and tissue characteristics were fused with live fluoroscopic imaging, however they were not visible for the implanting cardiologist. Therefore, this group, in whom we performed treatment planning but no real-time image-guidance, is further mentioned as the non-target group. In step 4 (six patients) *all* aforementioned tissue characteristics were determined and displayed in conjunction with live fluoroscopy in the catheterization theatre during LV lead implantation. Thus enabling the implanting cardiologist to perform image-guided LV lead placement in a targeted treatment group (target group).

In all patients implantation characteristics (radiation dose, procedure and fluoroscopy time, and peri-procedural complications) were collected together with electrical properties at the stimulation electrode (pacing threshold, LPN stimulation threshold, paced QRS duration and the electrical delay, which was measured as the interval from Q on the surface ECG to local sensing at the LV electrogram (QLV), divided by QRS duration (QLV/QRS). Echocardiography was performed before and 6 months after implantation to determine the presence of LV reverse remodeling (defined as a ≥ 15% reduction in LV end-systolic volume).

### Cardiac magnetic resonance imaging

CMR was performed 1–7 days prior to CRT implantation in all patients, using a 1.5 T Philips Ingenia scanner (Philips Healthcare, Best, The Netherlands). Gold standard late gadolinium enhancement (LGE) CMR scans were made to determine the size and the location of the myocardial scar. Short axis steady-state-free-precession cine images were made to determine areas of latest contraction, using feature tracking (CMR-FT) software (TomTec Arena, 2D Cardiac Performance Analysis MR, Version 1.2, Unterschleissheim, Germany). The settings during the CMR acquisitions were as follows. Cine: repetition time/echo time = 3.4 ms/1.7 ms, flip angle = 60°, voxel size = 1.67 × 1.67 mm, field of view = 32 × 32 cm, 192 × 192 matrix, 8 mm slice thickness, 30 phases/R–R interval, electrocardiogram-gated. LGE: repetition time/echo time = 3.2 ms/1.6 ms, flip angle = 15°, voxel size = 2.1 × 2.1 mm, field of view = 46 × 46 cm, 220 × 220 matrix, 8 mm slice thickness. Cine and LGE scans were made at the same positions with the same orientation.

### Cardiac computed tomography

CT scans were performed 2–14 days prior to CRT implantation in nine patients for the identification of LPN and CSO. CT images were acquired using Turbo Flash in a Siemens Somatom Force 384 (2 × 192) row scanner (Siemens Healthcare, Forchheim, Germany). The CT protocol was optimized for visualization of contrast in the venous system using a double bolus technique to ensure opacification of the CSO. The first bolus of 60 ml of iodinated contrast medium (saline:contrast ratio: 1:2, 300 mg of iodine/ml, Ultravist; Bayer AG, Berlin, Germany) was administered at the start and the second bolus of 80 ml was injected after 40 s. Both boluses were injected at a rate of 6 ml/s into the basilic vein. CT scanning was triggered by using a bolus-tracking technique, with the region-of-interest placed in the descending aorta. Image acquisition started 11 s after the attenuation reached the predefined threshold of 200HU. Scanning time was approximately 0.25 s. The reference tube potential and tube current were set to 100 kV and 350mAs, respectively. Both were regulated by automatic potential and tube current programs (Care kV and Care dose 4D). Images were reconstructed with a 1.0 mm slice thickness and a 0.4 × 0.4 mm pixel spacing with a Bv40d reconstruction kernel.

### Image processing

**Fig. 2:**
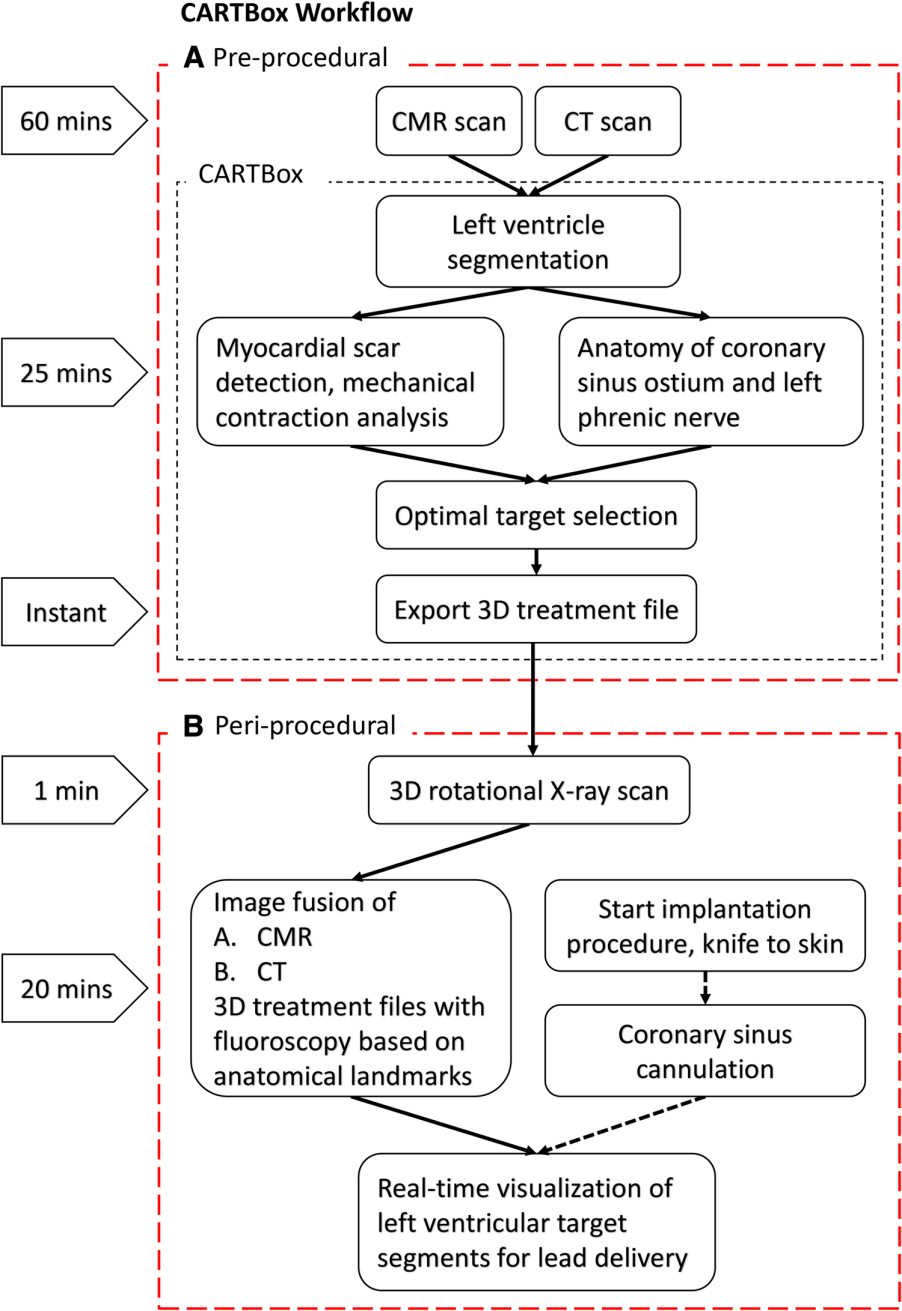
CARTBox workflow and time requirement. The pre-procedural workflow **a** consists of the acquisition of cardiac MRI and CT (60 min in total), and image processing in CARTBox. The image processing, required to identify the optimal site for LV stimulation, and necessary to produce a detailed 3D-model of the heart, takes approximately 25 min per scan. The implantation procedure **b** starts with acquiring a 3D-rotational X-ray scan (minutes). The 3D-treatment files are then semi-automatically fused with the 3D-rotational scan based on anatomy landmarks. This takes approximately 20 min and can be performed during RV lead implantation and coronary sinus cannulation. Using this approach, LV target areas can be visualised on live fluoroscopic images during LV lead implantation. *CMR* cardiac magnetic resonance, *CT* computed tomography

**Fig. 3:**
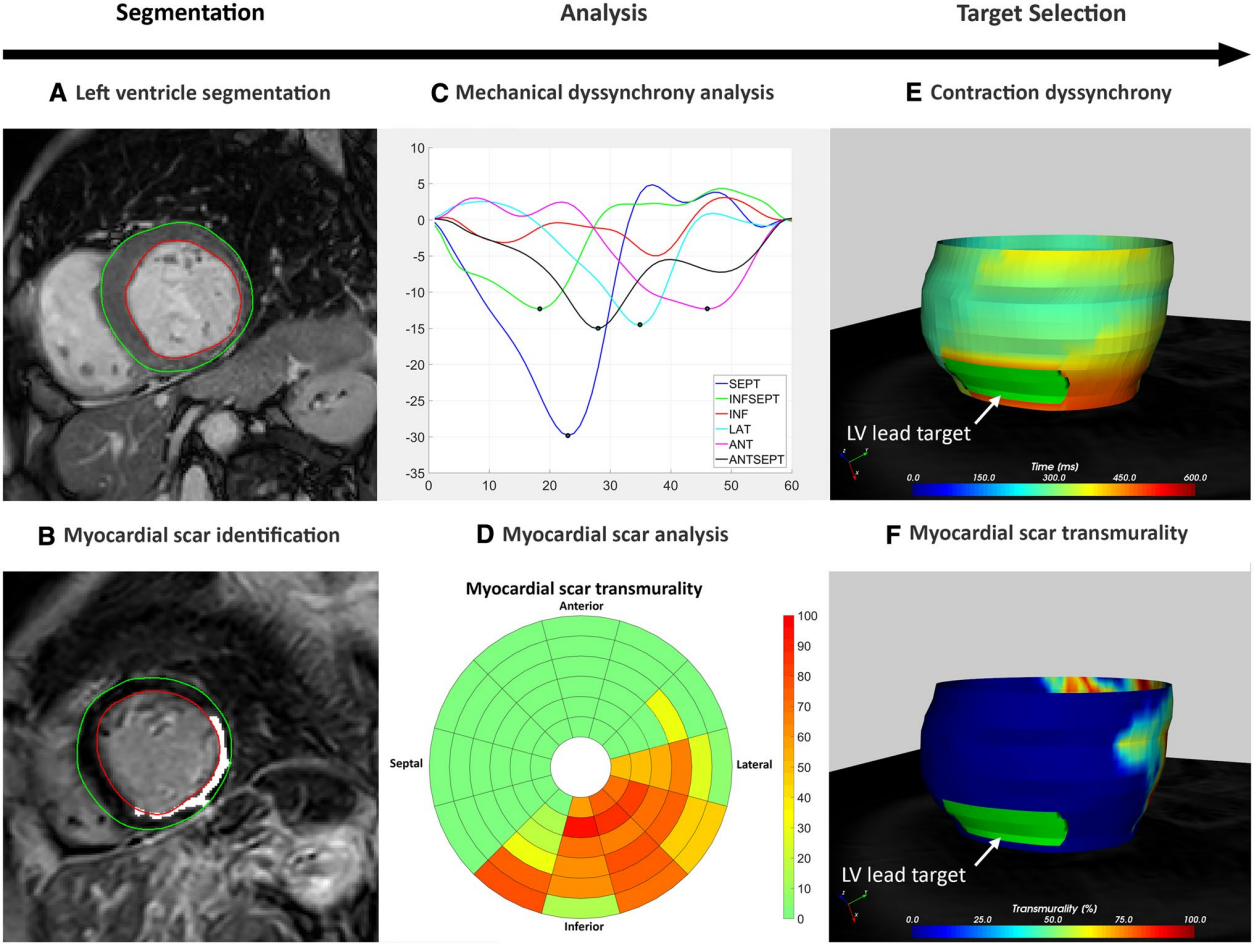
CARTBox workflow in images. **a** Segmentation of left ventricle. **b** Myocardial scar detected on CMR LGE scans. **c** Contraction timing analysis displaying delayed contraction of anterior and lateral segments. **d** Transmurality of scar showing inferolateral infarct of the left ventricle. **e**, **f** 3D-model of contraction timing (**e**) and scar transmurality (**f**) with manual selected target segment (green). *ANT* anterior, *ANTSEPT* anteroseptal, *INF* inferior, *INFSEPT* inferoseptal, *LAT* lateral, *LV* left ventricle, *SEPT* septal

Image processing with CARTBox consisted of three steps (Figs. 2, 3). The first step consisted of the segmentation of (a) scarred myocardium and dyssynchrony on CMR images, and (b) the identification of the LPN and CSO on CT-scans.

- To start, the LV endo- and epicardium of the end-diastolic short axis CMR cine and LGE images were automatically segmented. The full width at half maximum method was used for the segmentation of myocardial scar on LGE images (Fig. 3b) [9]. Segmentations were manually adjusted if necessary. For detection of the latest mechanical contracting segments, time to peak analysis was performed on the short axis CMR cine images using CMR-FT software [10, 11]. Time to peak endocardial circumferential strain was used for the identification of latest contracting segments (Fig. 3c) because circumferential strain is believed to produce higher intra- and interobserver reproducibility than segmental radial strain analysis [12, 13]. After image processing, scar transmurality and contraction timing data were projected on a 3D-epicardial surface mesh (Fig. 3e, f).
- After CMR processing, the location of the CSO and course of the LPN were segmented manually from CT data. A board-certified cardiac radiologist (FMH) reviewed the results of the segmentation processes.

In the second step, the implanting cardiologist selected the optimal area for LV lead delivery based on the course of the LPN and the 3D-CMR surface mesh containing scar transmurality [%] and contraction timing [ms] data (Fig. 3e, f). Optimal pacing sites were chosen in an area with 0% scar transmurality and most delayed contraction. Septal segments were excluded as target areas.

**Fig. 4:**
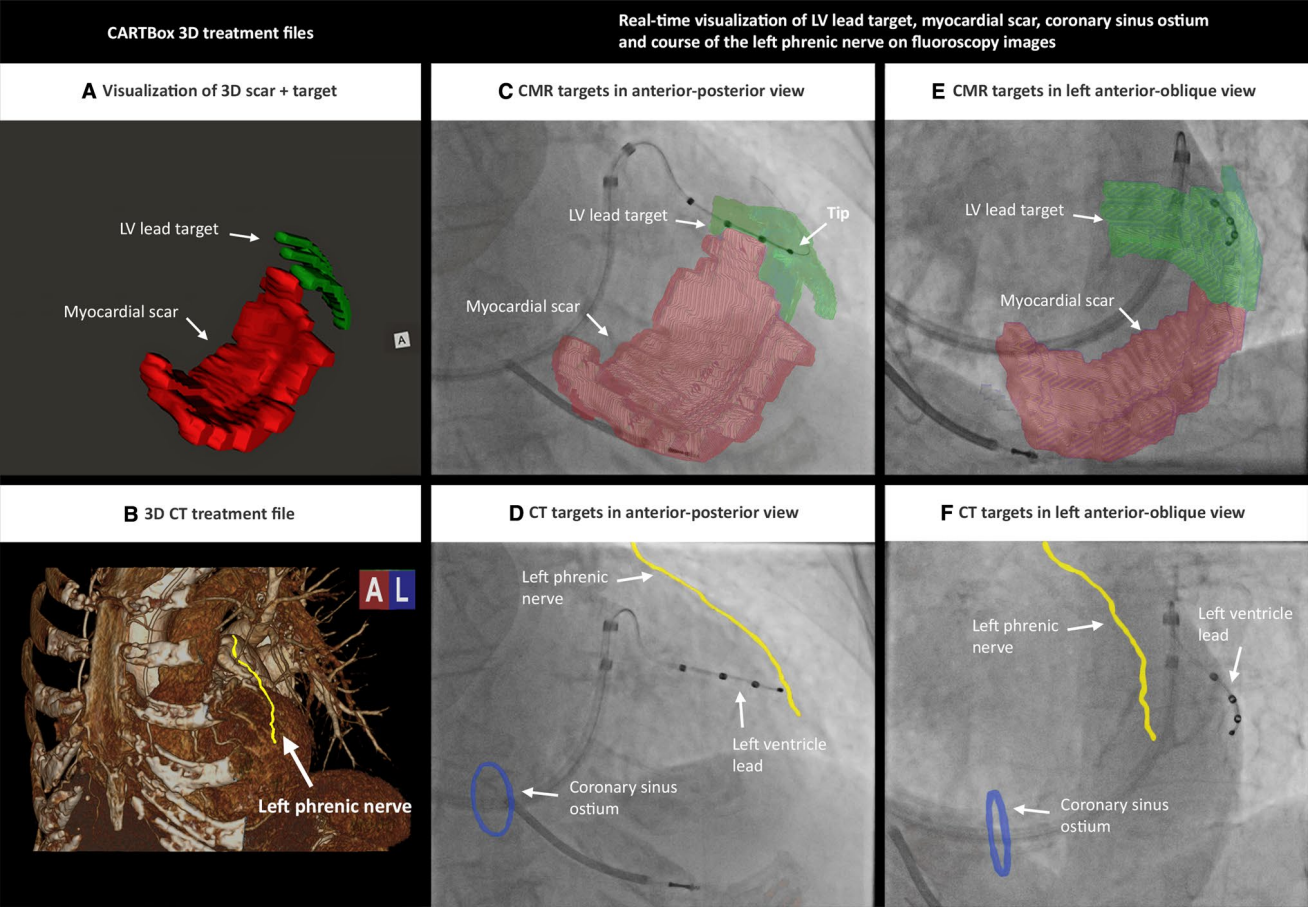
Real-time visualization of CMR and CT targets. **a**, **b** 3D-treatment file of CMR data (**a**) and CT data (**b**). **c**–**f** After 3D image fusion of the 3D-treatment dataset with fluoroscopy, the LV lead targets and scar segments (**c, e**) together with left phrenic nerve and coronary ostium (**d**, **f**) are visualized on live fluoroscopy during the LV lead implantation. *CMR* cardiac magnetic resonance, *CT* computed tomography

In the final step, two 3D-treatment files were created by CARTBox in the standard DICOM format. One 3D-treatment file contained myocardial scar transmurality and the LV target segments (Fig. 4a). The other 3D-treatment file consisted of the anatomy of the LPN and CSO (Fig. 4b).

### Image fusion

Prior to CRT implantation, a 3D-rotational scan was made in the catheterization theatre. In a single gantry rotation of 200°, a 3D CT-like dataset is acquired by using the Siemens Artis Zee (Syngo X workplace version B21), which allows 3D fusion of CMR and CT images with live fluoroscopy. After fusing the 3D-treatment datasets, 3D representations of the specific anatomical aspects (i.e. myocardial scar, LV lead target, LPN and/or CSO) were visualized in conjunction with the fluoroscopy images by assigning a unique color for each anatomic structure (Fig. 4). After registration the targets rotate accordingly upon rotation of the C-arm. The fused images were shown in a separate part of the screen in the catheterization theatre. Directly after CRT device implantation, a second 3D-rotational scan was acquired to measure the distances between the final LV pacing electrode location and the locations of the scar, LV lead target area, and LPN.

### CRT implantation

CRT device implantation was performed transvenously under local anesthesia. The right atrial and right ventricular leads were placed at conventional locations in the right atrium appendage and the right ventricular apicoseptal segment, respectively. After CS cannulation and coronary venous angiogram, a quadripolar LV lead was placed in one of the coronary veins overlying the LV free wall. After LV lead placement, all leads were connected to a CRT device.

### Statistical analysis

Statistical analysis was performed with SPSS (SPSS statistics 23.0, IBM, New York, USA). Each variable was tested for normality with a Shapiro–Wilk test. Continuous variables with a Gaussian distribution were described using mean, standard deviation and those with non-normal distribution were described with the median, interquartile range (IQR). Categorical data were described by an absolute number of occurrences and associated frequency (%). Differences between groups were assessed using nonparametric testing with Mann–Whitney U test for continuous data with non-normal distribution, and unpaired Student’s *t-*test for continuous variables with a Gaussian distribution. Pearson Chi-Square test was used for dichotomous variables. A *p*-value below 0.05 was considered significant.

## Results

Fifteen patients, of whom baseline characteristics are provided in Table 1, underwent de novo CRT implantation with a quadripolar LV lead. Patients were aged 68 ± 11 years, ten were male, eleven had a left bundle branch block, and seven had ischemic cardiomyopathy (ICM) with a mean scar burden of 21 ± 13% (Table 1). Median size of the pre-procedurally defined LV target area was 10% [6–11] of total LV surface. In all patients, CARTBox was successfully applied and merging of the treatment file with live fluoroscopy images did not lengthen the procedure. Merging was performed during pocket preparation and right ventricular lead implantation. Image fusion took an average of 14 ± 4 min for merging of the CT-scan, and 10 ± 6 min for merging the CMR scan. CSO visualization was successful in all patients that received a pre-procedural CT and had an overall fair agreement with the CSO at fluoroscopy images. There were no intra- or post-operative complications and no reported adverse effects on renal function.

**Table 1:** Demographic data

	All patients (n = 15)	Target group (n = 6)	Non-target group
Male gender (%)	10 (67)	3 (50)	7 (78)
Age (years)	68 ± 11	67 ± 13	69 ± 9
Body Mass Index (kg/m^2^)	26 ± 5	27 ± 6	26 ± 4
NYHA functional class (n, %)			
II	12 (80)	5 (83)	7 (78)
III	3 (20)	1 (17)	2 (22)
Left bundle branch block (n, %)^a^	11 (73)	5 (83)	6 (67)
QRS duration (ms)	162 ± 23	165 ± 26	160 ± 22
PR interval (ms)	188 ± 34	164 ± 27^†^	203 ± 29^†^
LV ejection fraction (%)	26 ± 5	27 ± 6	25 ± 5
LV end diastolic volume (ml)	209 [165–250]	175 [142–216]	222 [184–327]
LV end systolic volume (ml)	149 [123–198]	128 [96–169]	162 [135–250]
Ischemic cardiomyopathy (n, %)	7 (47)	2 (33)	6 (67)
Scar burden (%)	18 [13–28]	30 [15–45]	18 [7–28]

Values are in mean ± SD, median [interquartile range] and n (%). Significant differences between groups (*p* < 0.05) are indicated with a^†^

*LV* left ventricular, *NYHA* New York Heart Association

^a^Definition according to Strauss criteria

### LV implantation characteristics

The implantation characteristics and outcome data of patients who received real-time image-guided LV lead placement (target group) are displayed in Table 2. Total and LV implantation duration in this group were 146 ± 38 min and 47 ± 18 min respectively, fluoroscopy time was 36 ± 15 min. CRT implantation duration and fluoroscopy times were not statistically different from recent historical controls (185 ± 40 min, *p* = 0.07, and 27 ± 12 min, *p* = 0.17 respectively). Total radiation dose was 6758 ± 4201 cGycm^2^, the radiation dose of the pre-implantation and post-implantation 3D-rotational scan were 1188 ± 262 cGycm^2^ and 1313 ± 333 cGycm^2^ (Table 2). The radiation dose of the CRT implantation without 3D-rotational scan was 5753 ± 3038 cGycm^2^.

**Table 2:** CRT implantation and follow up characteristics

	Target group (n = 6)	Non-target group (n = 9)	p-value
Distance to target sites			
Distance to target (mm)	8 [0–22]	26 [17–46]	0.04
Distance to infarct (mm)	22 [21–23] (n = 2)	26 [14–51]	0.51
Distance to left phrenic nerve (mm)	44 [18–54]	44 [36–n/a]	0.61
Implantation characteristics			
Implantation duration (min)	146 ± 38	127 ± 35	0.38
LV lead implantation duration (min)	47 ± 18	55 ± 28	0.57
Fluoroscopy time (min)	36 ± 15	28 ± 12	0.30
Total radiation dose (cGycm^2^)	6758 ± 4201	8242 ± 6446	0.70
Pre-procedural 3D-angiogram radiation (cGycm^2^)	1188 ± 262	1449 ± 452	0.41
Post-procedural 3D-angiogram radiation (cGycm^2^)	1313 ± 333	1491 ± 439	0.57
Radiation dose CRT only (cGycm^2^)	5753 ± 3038	5303 ± 5847	0.91
LV lead electrical properties			
Paced QRS duration (ms)	153 ± 22	170 ± 22	0.18
Decrease QRS duration (ms)	− 12 ± 13	− 9 ± 27	0.10
Pacing threshold (V)	0.65 ± 0.39	0.58 ± 0.20	0.64
QLV (ms)	150 ± 8	130 ± 30	0.23
Ratio QLV/QRS (%)	85 ± 10	81 ± 16	0.66
Echocardiographic follow up			
LV end-systolic volume change (%)	− 30 ± 10	− 19 ± 19	0.28
LV ejection fraction change from baseline (%)	15 ± 5	10 ± 12	0.30

Values are in mean ± SD, median [interquartile range]

*LV* left ventricular, *QLV* interval from Q on the surface ECG to local sensing at the LV electrogram

### Results of image-guided CRT implantation

In all patients that received real-time image guidance, LV leads were placed out of scar, away from the LPN and within, or in close proximity to the CMR defined target area. In three out of six patients the LV lead was implanted within the target segment, in the other three patients, the LV lead was placed adjacent to the target area. In patients from the target group, LV leads were placed significantly closer to the target area compared to patients from the non-target group (8 mm [IQR 0–22] vs 26 mm [IQR 17–46], p = 0.04), while distance of the LV lead to scar and the LPN did not differ between groups. The electrical properties in the target group did not vary from the non-target group and they were as follows: mean pacing thresholds: 0.65 ± 0.39V vs 0.58 ± 0.20V, paced QRS duration: 153 ± 22 ms versus 170 ± 22 ms, change in QRS duration from baseline: − 12 ± 13 ms versus − 9 ± 27 ms, and QLV: 150 ± 8 ms versus 130 ± 30 ms (QLV/QRS ratio 85 ± 10% vs 81 ± 16) (Table 2).

At 6 months follow up, all patients from the target group showed echocardiographic response to CRT with a mean LVESV change of − 30 ± 10% and a mean LVEF improvement of 15 ± 5%.

## Discussion

This study demonstrates the feasibility of multimodality image fusion, for treatment planning and real-time image-guided LV lead delivery towards optimal pacing sites during CRT implantation procedures. Optimal pacing sites were pre-procedurally identified on CMR (i.e. latest contracting segment and scar location) and CT scans (i.e. anatomy of the LPN and CSO) and were intra-procedurally fused with live fluoroscopic projections. This allowed the implanting cardiologist to place the LV lead out of scar, away from the LPN and closer to the CMR defined target area compared to CRT implantation without real-time image-guidance, while implantation duration and fluoroscopy time were not increased compared to historical controls.

### Targeting LV lead towards predefined optimal pacing sites

Previous work demonstrated significantly more LV reverse remodeling, lower cardiac mortality and fewer heart failure hospitalizations in patients paced from within a target segment with significant electrical or mechanical delay [2, 3, 6, 14]. Measuring the QLV is a relatively simple technique for assessing LV activation delay, however, it provides limited information of total LV electrical activation because usually measurements are only performed at the LV anatomical target region. CMR-scans on the other hand can provide detailed information with regards to mechanical dyssynchrony and myocardial scar location. This supports the role of CMR for image-guided LV lead delivery in patients undergoing CRT implantations. Yet, only two previous studies established real-time visualization of target areas on fluoroscopy images during CRT implantation [15, 16]. Using a similar approach to our study, both studies showed the feasibility of real-time image-guided LV lead implantation. Importantly, they did not assess the course of the LPN, moreover, they assessed latest contracting segments by an automatic segmentation algorithm differentiating between the myocardium and the blood pool and in doing so assessed the time to minimum segmental endocardial volume. In our study, we used CMR-FT, the CMR equivalent of speckle-tracking echocardiography for contraction timing analysis. CMR-FT is a relatively easy technique for myocardial contraction timing analysis since the cine images are obtained during standard cardiac imaging protocols [6, 17]. In validation studies CMR-FT showed good agreement with CMR-tagging, the gold standard technique for the non-invasive assessment of myocardial deformation which requires separate acquisition of images [13, 18].

In the present study, real-time image-guided LV lead implantation enabled placing the LV lead closer to the target segment compared to LV lead implantation without real-time image-guidance (treatment planning only). LV lead delivery within a pre-procedurally defined target segment, however, remains challenging. We were able to place three out of six LV leads within the CMR target segment using the overlay with fluoroscopy. The lack of a suitable coronary vein at the target site can be an important factor that may prevent LV lead delivery to a target segment. Additionally, in the present study, we did not adhere to the American Heart Association (AHA) 17-segment to determine LV lead target segments [19], but we performed the data processing into smaller LV segments and allowed the cardiologist to freely choose a subset of the LV segments to construct a well visible LV target area. Data processing into smaller segments allows for a more precise delineation of scar tissue, and subsequently, more precise target area definition. Placing the LV within a smaller target segment, however, is more challenging. Real-time image-guidance enabled us to place the LV lead as close to the target site as possible in all patients. Whether more precise targeting leads to improved CRT outcomes needs yet to be determined.

### Limitations and challenges

This study was designed to demonstrate the feasibility of a novel treatment guidance platform for real-time visualization of optimal pacing sites during CRT implantation. Because the study was not designed or powered to demonstrate the superiority of real-time image-guided LV lead placement, no definite conclusions can be drawn regarding outcome data on implantation characteristics and end points. While, all patients that received real-time image-guided LV lead implantation were echocardiographic responder at follow up, other important factors probably have attributed to the high rate of reverse remodeling in patients from the target group. For instance, patients with persistent atrial fibrillation were excluded from study participation and patients from the target group had relatively favorable patient characteristics (e.g. more frequently LBBB, non-ICM and lower intracardiac volumes).

Furthermore, we recognize that a relatively high radiation dose was used due to the additional 3D-rotational scans and pre-implantation CT imaging. The radiation dose of the CRT implantation without 3D-rotational scans was comparable to previous work [20, 21]. In the present study, 3D-rotational scans were acquired before and after CRT implantation, however for visualization of optimal pacing sites onto fluoroscopic projections, performing a single 3D-rotational scan is sufficient. This adds approximately 20% of radiation dose to a CRT implantation. Detrimental effects of radiation, occur at a dose area product larger than 40,000 cGycm^2^ (assuming an effective radiation area of 100 cm^2^) [20] whereas, the threshold dose for skin erythema is 20,000 cGycm^2^ [21]. Using CARTBox, in the present study radiation dose thus remained well below the above-mentioned thresholds. In addition, performing a CT-scan is not standard care in patients undergoing CRT device implantation and is associated with increased cost and a slightly increased healthcare risk due to ionizing radiation (average 20–80 cGycm^2^) and the use of an iodinated contrast agent. Iodinated contrast agents may cause kidney dysfunction, especially in patients with pre-existing renal impairment. According to large, epidemiologically representative patient populations with chronic heart failure, about 10% of patients will have severe renal dysfunction (GFR < 30 ml min·1.73 m^2^) [22]. Therefore, performing both a CT scan and a CRT implantation may not be feasible in all patients eligible for CRT. Although the CARTBox platform can easily be implemented using CMR only, we chose to implement the CT scan in the current study, because visualizing the course of the LPN and CSO before implantation could potentially simplify CS cannulation, prevent LPN stimulation and accompanied LV lead relocation, and consequently, reduce implant times. Pre-procedural detailed evaluation of the coronary venous anatomy on CT images could take the concept for targeted LV lead implantation even further. However, the timing of the intravenous contrast administration to the venous phase is not a standard procedure and is especially difficult in heart failure patients. Furthermore, even when performed optimally, it does not permit the visualization of the smaller venous branches. Therefore in the present study, we did not evaluate the CS anatomy preoperatively on CT, but chose to use the CS venogram instead, which is acquired during CRT implantation and which is the current standard to visualize complete CS anatomy. Importantly, emerging technologies in CMR and CT scan protocols and image analysis algorithms (i.e. detection of myocardial scar and dyssynchrony on cardiac CT [23] and the anatomy of the LPN and coronary sinus on CMR [24]) could in the near future negate the necessity for both pre-procedural CMR and CT. This would especially be of value in patients with a contra-indication for CMR or CT, such as patients with impaired renal function, claustrophobia, documented allergy to gadolinium, or patients with non-MRI conditional devices.

### Future implications

Despite the aforementioned challenges, and based on the superior patient outcomes of targeted LV lead placement, demonstrated by previous studies [2, 3, 6, 15], we believe that the technology of real-time image-guided LV lead implantation towards optimal pacing sites is clinically promising. Technical advancements such as the development of the snare technique and octopolar LV leads, together with other pacing techniques, such as endocardial pacing, will probably allow for a more precise delivery of LV leads into predefined, smaller, target areas. Technologies that enable visualization of optimal pacing sites, therefore, may become of high value for implanting physicians. Moreover, further developments of CARTBox have enabled the use of merging the 3D-CMR or CT treatment dataset with standard fluoroscopy set-ups to omit the need for a 3D-rotational scan. This reduces ionizing radiation, and increases the uptake of the technology in CRT implanting hospitals.

## Conclusions

Real-time image-guided LV lead placement by fusion of CMR- and CT-images with fluoroscopy images during CRT device delivery is feasible and endorses placing the LV lead closer to the target segment and out of scar compared to treatment planning only. Merging of target segments on to live fluoroscopy can be performed rapidly without prolongation of procedure time. Further investigation of this technology in clinical practice with larger patient cohorts is necessary to determine whether real-time image-guided LV lead delivery leads to improved patient outcomes and whether this approach is cost effective.

## Abbreviations

3D: Three dimensional
AHA: American Heart Association
CMR: Cardiac magnetic resonance imaging
CMR-FT: Cardiac magnetic resonance feature tracking
CRT: Cardiac resynchronization therapy
CSO: Coronary sinus ostium
CT: Computed tomography
ICM: Ischemic cardiomyopathy
LGE: Late gadolinium enhancement
LPN: Left phrenic nerve
LV: Left ventricular
QLV: Interval from Q on the surface ECG to local sensing at the LV electrogram
